# Immediate activation of chemosensory neuron gene expression by bacterial metabolites is selectively induced by distinct cyclic GMP-dependent pathways in *Caenorhabditis elegans*

**DOI:** 10.1371/journal.pgen.1008505

**Published:** 2020-08-10

**Authors:** Jaeseok Park, Joshua D. Meisel, Dennis H. Kim

**Affiliations:** 1 Division of Infectious Diseases, Boston Children’s Hospital, and Department of Pediatrics, Harvard Medical School, Boston, Massachusetts, United States of America; 2 Department of Biology, Massachusetts Institute of Technology, Cambridge, Massachusetts, United States of America; Brown University, UNITED STATES

## Abstract

Dynamic gene expression in neurons shapes fundamental processes in the nervous systems of animals. However, how neuronal activation by different stimuli can lead to distinct transcriptional responses is not well understood. We have been studying how microbial metabolites modulate gene expression in chemosensory neurons of *Caenorhabditis elegans*. Considering the diverse environmental stimuli that can activate chemosensory neurons of *C*. *elegans*, we sought to understand how specific transcriptional responses can be generated in these neurons in response to distinct cues. We have focused on the mechanism of rapid (<6 min) and selective transcriptional induction of *daf-7*, a gene encoding a TGF-β ligand, in the ASJ chemosensory neurons in response to the pathogenic bacterium *Pseudomonas aeruginosa*. DAF-7 is required for the protective behavioral avoidance of *P*. *aeruginosa* by *C*. *elegans*. Here, we define the involvement of two distinct cyclic GMP (cGMP)-dependent pathways that are required for *daf-7* expression in the ASJ neuron pair in response to *P*. *aeruginosa*. We show that a calcium-independent pathway dependent on the cGMP-dependent protein kinase G (PKG) EGL-4, and a canonical calcium-dependent signaling pathway dependent on the activity of a cyclic nucleotide-gated channel subunit CNG-2, function in parallel to activate rapid, selective transcription of *daf-7* in response to *P*. *aeruginosa* metabolites. Our data suggest that fast, selective early transcription of neuronal genes require PKG in shaping responses to distinct microbial stimuli in a pair of *C*. *elegans* chemosensory neurons.

## Author summary

The nervous systems of animals carry out the crucial roles of sensing and responding to the external environment. When the free-living microscopic roundworm *C*. *elegans* is exposed to the pathogenic bacteria *Pseudomonas aeruginosa*, sensory neurons detect metabolites produced by the pathogen and induce expression of the gene for a neuroendocrine ligand called DAF-7. In turn, activity of DAF-7 is required for the avoidance response to *P*. *aeruginosa*, allowing the animals to survive longer. Here, we genetically dissect the signal transduction pathways between the sensation of *P*. *aeruginosa* metabolites and the expression of *daf-7* in a pair of *C*. *elegans* sensory neurons. We show that the intracellular signaling molecule cyclic-GMP is a key signaling intermediate. In addition, we show that there are calcium-dependent and calcium-independent pathways that are both required to engage *daf-7* expression, highlighting an organizational principle that allows neurons to distinguish between various stimuli.

## Introduction

Chemosensory systems of animals transduce external chemical stimuli into neuronal signals, with diverse roles in animal physiology [1–3]. A challenge for chemosensory systems is detecting and processing a diverse set of environmental information to generate appropriate neuronal and behavioral responses. Whereas neurons utilize electrical impulses in rapid data transmission, changes in gene expression serve as a mechanism for transducing information over a longer time scale. Activity-dependent transcription of immediate-early genes has been shown to involve the activation of calcium-dependent signal transduction converging on CREB [4]. Our recent work has focused on understanding how changes in gene expression in chemosensory neurons of *C*. *elegans* can be modulated by interactions with its microbial environment and internal cues [5–7].

Interactions with microbes, in a number of forms such as parasitism, symbiosis, predation, and exploitation, have shaped the evolution of animals. There has been an increasing appreciation for the role of the nervous system in recognizing and responding to microbes in the environment.

Disgust, for example in response to rotting food, elicits avoidance behavior [8]. At the cellular level, examples include host nociceptive neurons that have been shown to respond to microbial toxins to regulate immune responses [9], and chemosensory tuft cells, which have recently been found to sense the gut environment using canonical G-protein pathways to mediate appropriate immune responses [10–12].

The nematode *C*. *elegans* is usually found in rotting organic material, a complex environment in which the animal has to navigate between bacterial food, pathogens, predators, competitors, and parasites [13]. With an expanded family of chemoreceptor genes in its genome and a limited set of chemosensory neurons that function to regulate diverse aspects of animal physiology, the chemosensory system of *C*. *elegans* enables navigation and ultimately survival in its predominantly microbial natural environment [14]. We investigated how the behavior of *C*. *elegans* is modulated by pathogenic bacteria, specifically *Pseudomonas aeruginosa*, a devastating opportunistic pathogen of humans that is commonly found in soil and water and can also kill *C*. *elegans* [15].

*C*. *elegans* exhibits an aversive learning behavior in response to exposure and infection by *P*. *aeruginosa* [16,17]. Pathogen avoidance behavior results from a modulation of internal state caused by intestinal infection, coupled with integration of innate recognition of *P*. *aeruginosa* [7]. We previously showed that the detection of virulence-associated secondary metabolites produced by *P*. *aeruginosa* can alter the neuronal expression pattern of *daf-7*, which encodes a TGF-β ligand that regulates diverse aspects of *C*. *elegans* physiology and is required for avoidance of *P*. *aeruginosa* [6]. Whereas *daf-7* was previously known to be only expressed in the ASI head chemosensory neurons, we showed that exposure to *P*. *aeruginosa* causes the rapid (within six minutes) accumulation of *daf-7* mRNA in the ASJ sensory neurons [6]. We also showed that exposure to the *P*. *aeruginosa* secondary metabolite phenazine-1-carboxamide induced an increase in calcium in the ASJ neurons. Considering that abiotic stimuli have previously been shown to increase calcium levels in the ASJ neurons [18–20], whereas induction of *daf-7* expression in the ASJ neurons is highly selective for *P*. *aeruginosa* metabolites, we sought to define the molecular determinants of the selective transcription of *daf-7* in the ASJ neurons in response to the innate recognition of virulence-associated secondary metabolites produced by *P*. *aeruginosa*.

Here, we have taken a genetic approach to identify and characterize the signal transduction pathways in the ASJ neurons that couple the sensing of *P*. *aeruginosa* metabolites to the induction of *daf-7* transcription. We define distinct, parallel calcium-independent and canonical calcium-dependent pathways, each of which are dependent on cGMP signaling, which converge to selectively activate *daf-7* expression in the ASJ neurons in response to *P*. *aeruginosa* metabolites.

## Results

### The cyclic nucleotide-gated channel subunit CNG-2 is required for *daf-7* expression in the ASJ neurons in response to *P*. *aeruginosa*

We previously described the characterization of mutants defective in the induction of *daf-7* expression in the ASJ neurons in response to *P*. *aeruginosa*: *gpa-2* and *gpa-3*, each encoding G protein alpha subunits*; tax-2* and *tax-4*, encoding components of a cyclic nucleotide-gated channel; and *daf-11*, encoding a receptor guanylate cyclase [6]. These data were suggestive of a chemosensory signal transduction cascade that functions in the induction of *daf-7* expression in the ASJ neurons in response to *P*. *aeruginosa* metabolites.

**Fig 1:**
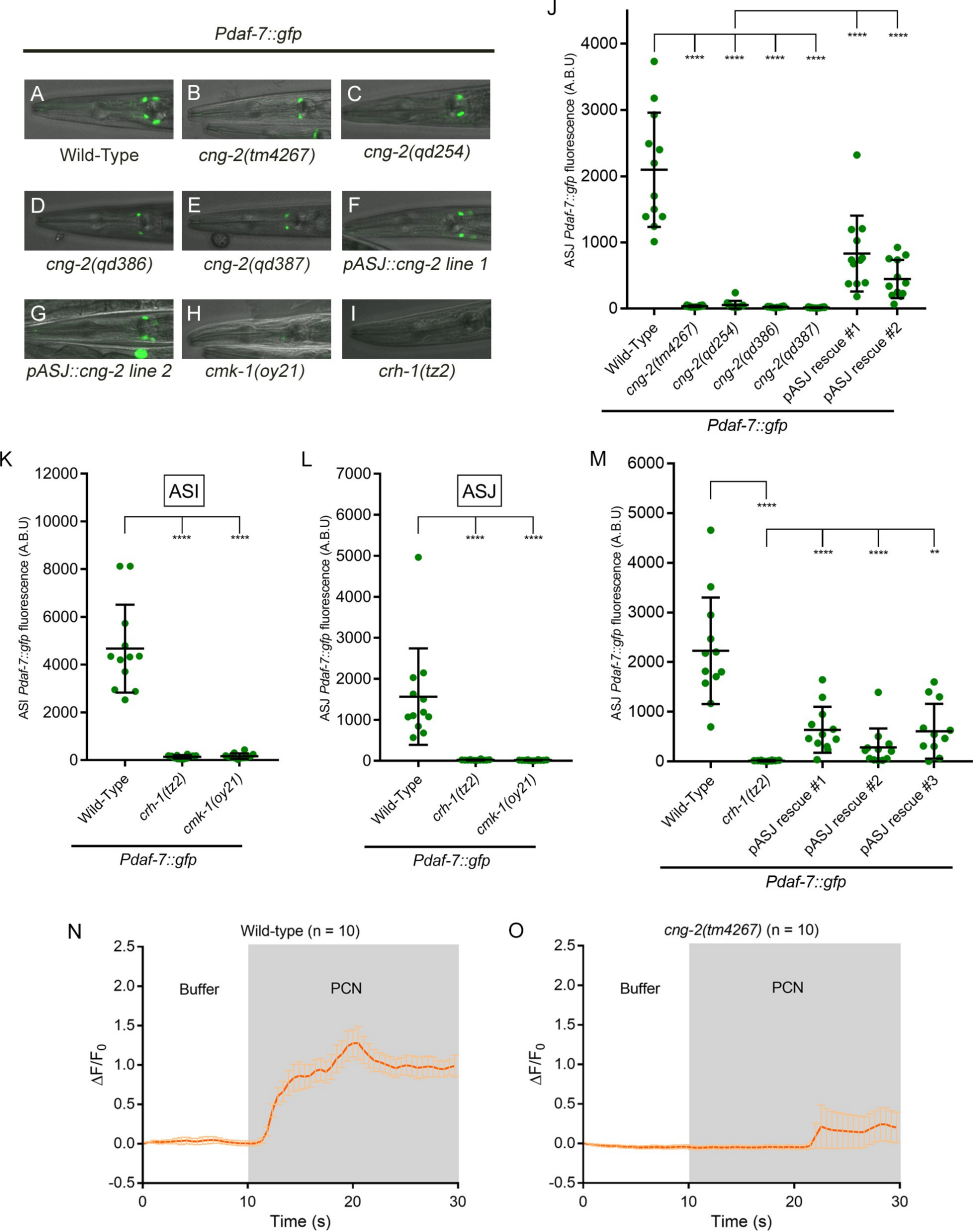
CNG-2 activates *daf-7* induction upon *P*. *aeruginosa* exposure in a calcium-dependent manner. (A-I) *Pdaf-7*::*gfp* expression after exposure to *P*. *aeruginosa* for various genotypes. (J) Maximum fluorescence values of *Pdaf-7*::*gfp* in ASJ neurons in various *cng-2* mutant backgrounds following *P*. *aeruginosa* exposure. Both rescue lines shown have the mutation *cng-2(qd254)* in the background. (K-L) Maximum fluorescence values of *Pdaf-7*::*gfp* in ASI and ASJ neurons in *crh-1* and *cmk-1* mutants following *P*. *aeruginosa* exposure. (M) Maximum fluorescence values of *Pdaf-7*::*gfp* in ASJ neurons of *crh-1* mutants expressing *crh-1* cDNA in ASJ neurons. All three rescue lines shown have the mutation *crh-1(tz2)* in the background. (N-O) GCaMP5 fluorescence change in the ASJ neurons of wild-type or *cng-2* mutant when exposed to buffer (DMSO) followed by 80 μg/ml phenazine-1-carboxamide (PCN). Error bars in J-M indicate standard deviation, and error bars in N-O indicate standard error of the mean. ****p < 0.0001, **p < 0.01 by Mann-Whitney U test.

We carried out further characterization of mutants isolated from a forward genetic screen that are defective in the induction of a *Pdaf-7*::*gfp* reporter transgene in the ASJ neurons in response to *P*. *aeruginosa*. We isolated an allele of *cng-2(qd254)* that has a splice-site mutation predicted to cause a frameshift, resulting in a non-functional protein product. This mutant showed no expression of *Pdaf-7*::*gfp* in the ASJ neurons in the presence of *P*. *aeruginosa*, which we further confirmed with additional putative null alleles of *cng-2*: *tm4267*, *qd386* and *qd387* (Fig 1A–1E and 1J). Unlike *tax-2* and *tax-4* mutants, *cng-2* animals did not differ from wild type in *Pdaf-7*::*gfp* expression in the ASI neurons (Fig 1A–1E, S1 Fig) [6]. Previous research indicate *cng-*2 is expressed in a number of head neurons, including the ASJ neurons [21]. Expression of *cng-2* cDNA specifically in the ASJ neurons using the ASJ-specific promoter *trx-1* was sufficient to rescue the *Pdaf-7*::*gfp* expression in the ASJ neurons (Fig 1F, 1G and 1J), suggestive that CNG-2 functions cell-autonomously in the ASJ neurons to mediate induction of *daf-7* transcription in response to *P*. *aeruginosa* infection.

We previously showed that exposure to the *P*. *aeruginosa* secondary metabolite, phenazine-1-carboxamide (PCN), results not only in the induction of *daf-7* expression in the ASJ neuron pair, but also in a rapid increase of calcium levels in the ASJ neurons [6]. The molecular identity of CNG-2 and relevant literature on the chemosensory apparatus in the ASJ neurons [14] led us to test whether CNG-2 might be an integral part of the cation channel that is responsible for the observed calcium influx. We observed that the influx of calcium ions in the ASJ neurons that is observed upon exposure of wild-type animals to the *P*. *aeruginosa* metabolite phenazine-1-carboxamide was abrogated in *cng-2* animals (Fig 1N and 1O).

We next examined mutants carrying mutations in the genes *cmk-1*, the *C*. *elegans* homolog of calcium/calmodulin-dependent kinase CaMKI/IV, and *crh-1*, the *C*. *elegans* homolog of the transcription factor CREB, and we observed that both genes are required for *daf-7* expression in the ASJ neurons (Fig 1H, 1I, 1K and 1L). ASJ-specific expression of *crh-1* conferred partial rescue (Fig 1M). However, we note that both *cmk-1* and *crh-1* mutants also showed minimal expression of *daf-7* in the ASI neurons (Fig 1H, 1I, 1K and 1L), raising the concern of pleiotropic effects on the development and/or physiology of the nervous systems of these mutants. Thus, our data, taken together with prior studies of CREB-dependent signaling, are consistent with a role for CRH-1 downstream of a calcium-dependent signaling pathway activated by CNG-2/TAX-2/TAX-4, but potential developmental pleiotropic effects in the analysis of these mutants represents an important limitation in our interpretation of these data.

### cGMP-dependent signal transduction activates *daf-7* expression in the ASJ neurons

**Fig 2:**
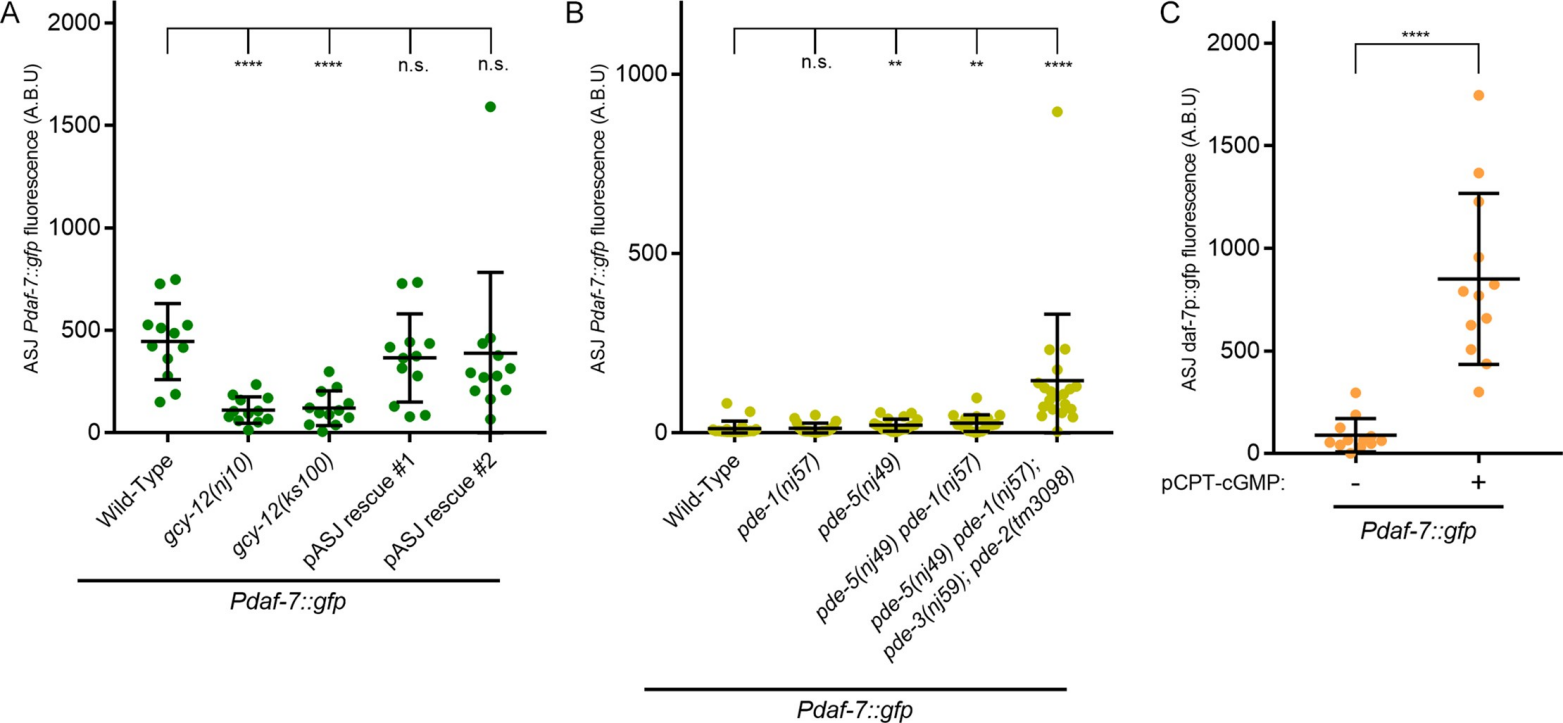
Elevation of cGMP levels is sufficient for the induction of *daf-7* in the ASJ neurons. (A) Maximum fluorescence values of *Pdaf-7*::*gfp* in ASJ neurons in various *gcy-12* mutant backgrounds following *P*. *aeruginosa* exposure. Both rescue lines shown have the mutation *gcy-12(ks100)* in the background. (B) Maximum fluorescence values of *Pdaf-7*::*gfp* in ASJ neurons of various phosphodiesterase (PDE) mutants. Animals were maintained on the *E*. *coli* strain OP50. (C) Maximum fluorescence values of *Pdaf-7*::*gfp* in ASJ neurons after exposure to 5 mM pCPT-cGMP; animals were maintained on the *E*. *coli* strain OP50. All error bars indicate standard deviation. ****p < 0.0001, **p < 0.01 by Mann-Whitney U test.

The requirements for components of a cyclic nucleotide-gated channel, CNG-2/TAX-2/TAX-4, and DAF-11, a guanylate cyclase, in the induction of *Pdaf-7*::*gfp* expression in response to *P*. *aeruginosa* metabolites suggested the involvement of cGMP-dependent signaling [6,14]. We demonstrated that mutations in the *gcy-12* gene, encoding another guanylate cyclase subunit, also caused markedly reduced *Pdaf-7*::*gfp* expression in the ASJ neurons in response to *P*. *aeruginosa*, but did not abolish *Pdaf-7*::*gfp* expression in the ASI neuron pair as was observed for the *daf-11* mutant (Fig 2A; S2 Fig) [6]. In addition, we observed that expression of the *gcy-12* cDNA specifically in the ASJ neurons rescued the induction of *Pdaf-7*::*gfp* expression in the ASJ neurons in response to *P*. *aeruginosa* (Fig 2A).

In order to investigate further the involvement of cGMP-dependent signaling in the induction of *daf-7* expression in the ASJ neurons, we examined the four *C*. *elegans* genes encoding phosphodiesterases (PDEs) that are predicted to cleave cGMP: PDE-1, PDE-2, PDE-3, and PDE-5 [22]. We observed that loss-of-function of all four PDEs resulted in the induction of *Pdaf-7*::*gfp* expression in the ASJ neurons even in the absence of *P*. *aeruginosa* (Fig 2B). Exposure of the quadruple mutant to *P*. *aeruginosa* resulted in a further increase of *Pdaf-7*::*gfp* transcription (S3 Fig). Mutations in only a subset of the genes encoding PDEs conferred a considerably weaker expression of *Pdaf-7*::*gfp* than the quadruple mutant (Fig 2B), suggestive that the PDEs function redundantly in the ASJ neurons. We also examined the effect of addition of a cell-permeable, non-hydrolysable analog of cGMP, pCPT-cGMP, to wild-type animals in the absence of *P*. *aeruginosa*, and we observed the marked induction of expression of *daf-7* in the ASJ neurons (Fig 2C).

### The cGMP-dependent protein kinase G EGL-4 upregulates *daf-7* expression in ASJ neurons in response to *P*. *aeruginosa*

The involvement of cGMP- and calcium-dependent signaling support a role for canonical activity-dependent signaling pathways in the induction of *daf-7* expression in response to *P*. *aeruginosa* metabolites. However, prior studies and our observations indicated that multiple stimuli including low pH, *E*. *coli* supernatant, sodium chloride and temperature changes can cause calcium influx in the ASJ neurons without the robust upregulation of *daf-7* observed in the presence of *P*. *aeruginosa* (S4 Fig) [18–20]. We sought to define additional, calcium-independent mechanisms that might be involved in the selective transcriptional response to *P*. *aeruginosa* metabolites.

**Fig 3:**
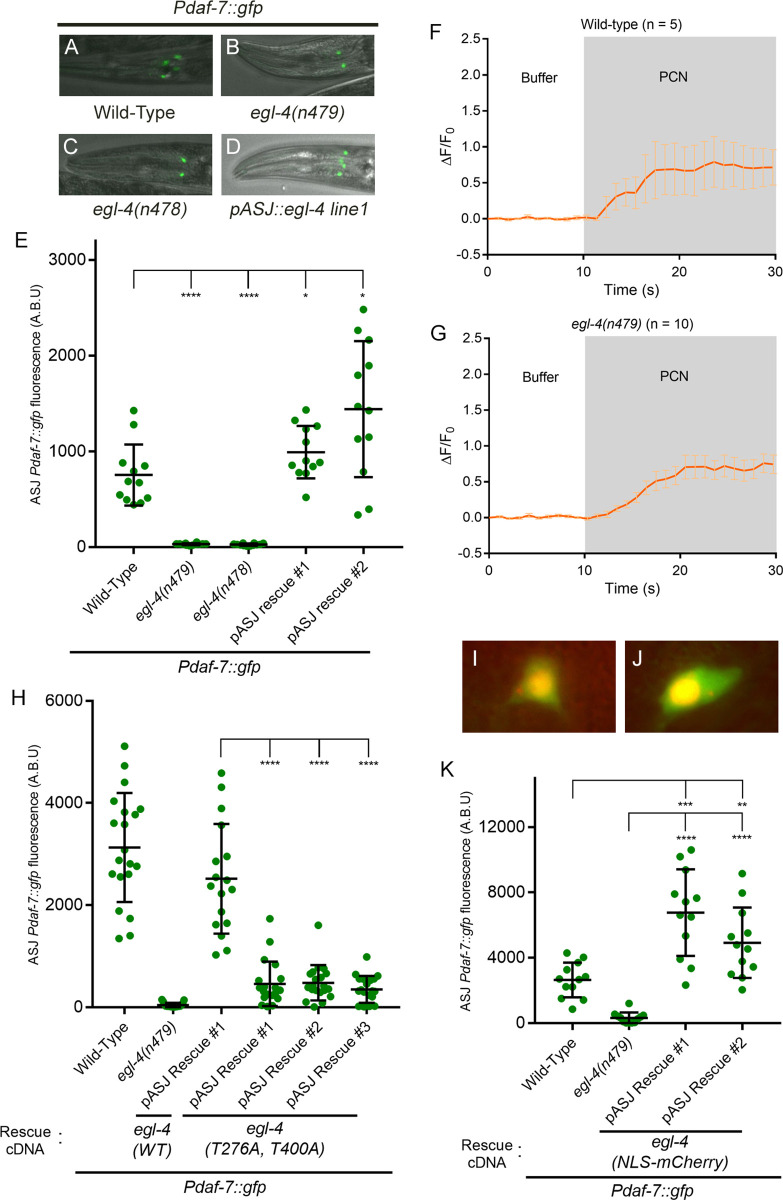
EGL-4 is required in the nucleus for the induction of *daf-7* in ASJ neurons upon *P*. *aeruginosa* exposure. (A-D) *Pdaf-7*::*gfp* expression after exposure to *P*. *aeruginosa* for various *egl-4* backgrounds. (E) Maximum fluorescence values of *Pdaf-7*::*gfp* in ASJ neurons in various *egl-4* mutant backgrounds following *P*. *aeruginosa* exposure. Both rescue lines shown have the mutation *egl-4(n479)* in the background. (F-G) GCaMP5 fluorescence change in the ASJ neurons when exposed to buffer (DMSO) followed by 66 μg/ml phenazine-1-carboxamide (PCN) in wild-type or *egl-4* mutants. (H) Maximum fluorescence values of *Pdaf-7*::*gfp* in ASJ neurons of *egl-4(n479)* mutants expressing WT *egl-4* cDNA or cGMP-binding defective (T276A, T400A) *egl-4* cDNA in the ASJ neurons. (I-J) NLS-mCherry-EGL-4 proteins are localized to the nucleus of ASJ neurons. NLS-mCherry-EGL-4 is seen in the red channels, and GFP from *Pdaf-7*::*gfp* is seen in the green channels. GFP is observed throughout the ASJ neurons, outlining the cells. (K) Maximum fluorescence values of *Pdaf-7*::*gfp* in ASJ neurons of *egl-4(n479)* mutants containing the NLS-mCherry-EGL-4 constructs following exposure to P. aeruginosa; the *egl-4(n479)* column data was from non-transgenic siblings of rescue line #1. Error bars in E, H and K indicate standard deviation, and errors bars in F and G indicate standard errors of the mean. ****p < 0.0001, ***p < 0.001, **p < 0.01, *p < 0.05 by Mann-Whitney U test.

**Fig 4:**
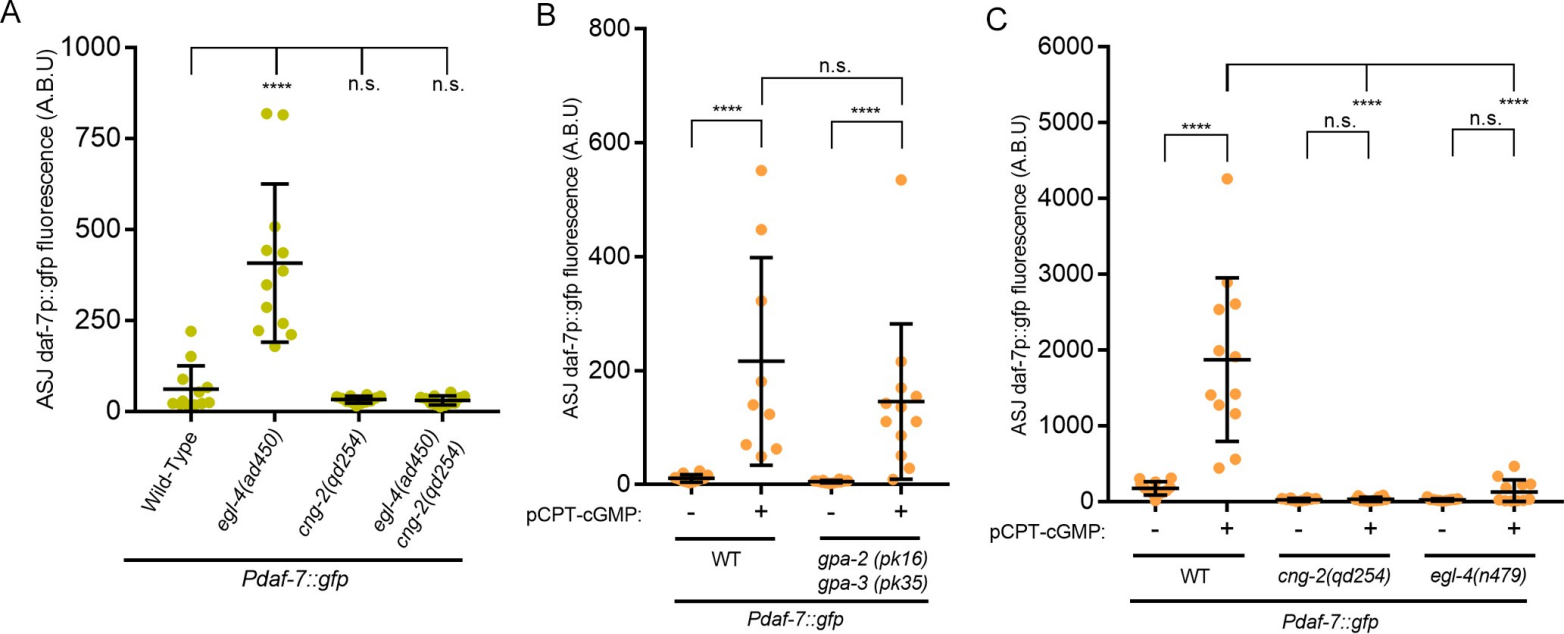
EGL-4 and CNG-2 work concurrently in parallel pathways downstream of cGMP to induce *daf-7*. (A) Epistasis analysis using *cng-2*(loss-of-function) and *egl-4*(gain-of-function) alleles. Animals were maintained on the *E*. *coli* strain OP50. (B-C) Maximum fluorescence values of *Pdaf-7*::*gfp* in ASJ neurons of various mutants following exposure to 5mM pCPT-cGMP; animals were maintained on the *E*. *coli* strain OP50. All error bars indicate standard deviation. ****p < 0.0001 by Mann-Whitney U test.

The dependence of *daf-7* expression in the ASJ neurons on cGMP led us to consider the involvement of the cGMP-dependent protein kinase G (PKG), EGL-4. EGL-4 has been implicated in various phenotypes including egg-laying behavior, chemosensory behavior, sleep-like state, satiety signaling, and aversive learning behaviors [23–33]. We observed that presumptive loss-of-function *egl-4(n478)* and *egl-4(n479)* mutants exhibited a lack of *Pdaf-7*::*gfp* expression in the ASJ neurons in response to *P*. *aeruginosa* (Fig 3A–3C and 3E). Expression of *egl-4* cDNA in the ASJ neurons was sufficient to rescue *daf-7* expression on *P*. *aeruginosa* (Fig 3D and 3E). A gain-of-function allele, *egl-4(ad450)*, exhibited detectable expression of *daf-7* expression in the ASJ neurons even in the absence of *P*. *aeruginosa* (Fig 4A). In order to examine whether EGL-4 functioned in a calcium-dependent or calcium-independent manner, we examined how *egl-4* loss-of-function affected the influx of calcium into the ASJ neurons observed upon exposure to phenazine-1-carboxamide. We found that unlike the *cng-2(tm4267)* mutant, the *egl-4(n479)* mutant showed a wild-type calcium level increase in ASJ neurons upon exposure to phenazine-1-carboxamide (Fig 3F and 3G).

Previous studies in the AWC neurons identified two critical cGMP-binding threonine residues in EGL-4 that are both necessary for EGL-4 function [26]. We sought to examine if cGMP-binding to EGL-4 is essential for *daf-7* transcription in ASJ in response to *P*. *aeruginosa* by constructing a mutant *egl-4* transgene in which the two aforementioned threonine residues were mutated to alanine. We observed that expression of this defective *egl-4* construct in the ASJ neurons of the *egl-4(n479)* mutant was unable to fully rescue the *daf-7* defect, in contrast with the wild-type rescue (Fig 3H).

PKGs have been reported to function in the regulation of gene expression, with activity associated with the nuclear translocation of PKGs [24,26,28,34]. We sought to examine the subcellular site of activity of EGL-4 in the regulation of *daf-7* expression. We first examined the expression of an mCherry::EGL-4 fusion construct, but we observed expression in both the nucleus and cytosol that did not detectably change with exposure to *P*. *aeruginosa*. We considered whether the diffuse expression might be the result of transgene overexpression, and thus we further examined two additional constructs, engineered to effect distinct subcellular localization. We expressed mCherry::EGL-4 with an additional nuclear localization sequence (NLS) at the N-terminus [26], and we confirmed that expression of this EGL-4-derived transgene was localized to the nucleus (Fig 3I and 3J). We observed that expression of this nuclear EGL-4 construct rescued the *daf-7* expression defect in the ASJ neurons (Fig 3K). Unfortunately, complementary experiments utilizing an mCherry::EGL-4 construct with the NLS deleted did not result in complete nuclear exclusion of the EGL-4-derived transgene, possibly due to transgene overexpression, precluding interpretation of corresponding rescue experiments (S5 Fig). These data are thus not definitive in defining the subcellular localization of EGL-4 in the regulation of *daf-7* expression but are consistent with a role for EGL-4 in the nucleus to regulate *daf-7* transcription in the ASJ neurons in response to *P*. *aeruginosa*.

To better define the respective roles of *cng-2* and *egl-4* by examining their genetic interactions, we again utilized the gain-of-function allele of *egl-4*, *ad450*. In the *egl-4(ad450) cng-2(qd254)* double mutant, the expression we observed in the *egl-4(ad450)* mutant was abolished (Fig 4A), which suggested that basal CNG-2-dependent calcium-dependent signaling in the absence of *P*. *aeruginosa* is required for the observed *daf-7* expression. We further sought to gain clarity regarding the pathway involving *cng-2* and *egl-4* by seeing how each of the mutants might change their *daf-7* expression in response to the addition of pCPT-cGMP. We first tested mutants of the heterotrimeric G-protein *gpa-2(pk16)* and *gpa-3(pk35)*. These proteins are thought to act in the initial steps of the chemosensory cascade by associating with the presumptive receptor for *P*. *aeruginosa* metabolites, and we have previously shown that the *gpa-2 gpa-3* double mutant is defective in *daf-7* transcription in response *to P*. *aeruginosa* [6]. Consistent with this prediction, adding pCPT-cGMP to *gpa-2 gpa-3* double mutants elicited the same induction of *Pdaf-7*::*gfp* expression in the ASJ neurons as observed in wild-type animals (Fig 4B). However, when pCPT-cGMP was added to *cng-2* and *egl-4* mutants, the response was markedly attenuated or absent (Fig 4C), consistent with roles for CNG-2 and EGL-4 functioning downstream of and dependent on a cGMP signal in the induction of *daf-*7 expression in the ASJ neuron pair in response to *P*. *aeruginosa* metabolites.

## Discussion

**Fig 5:**
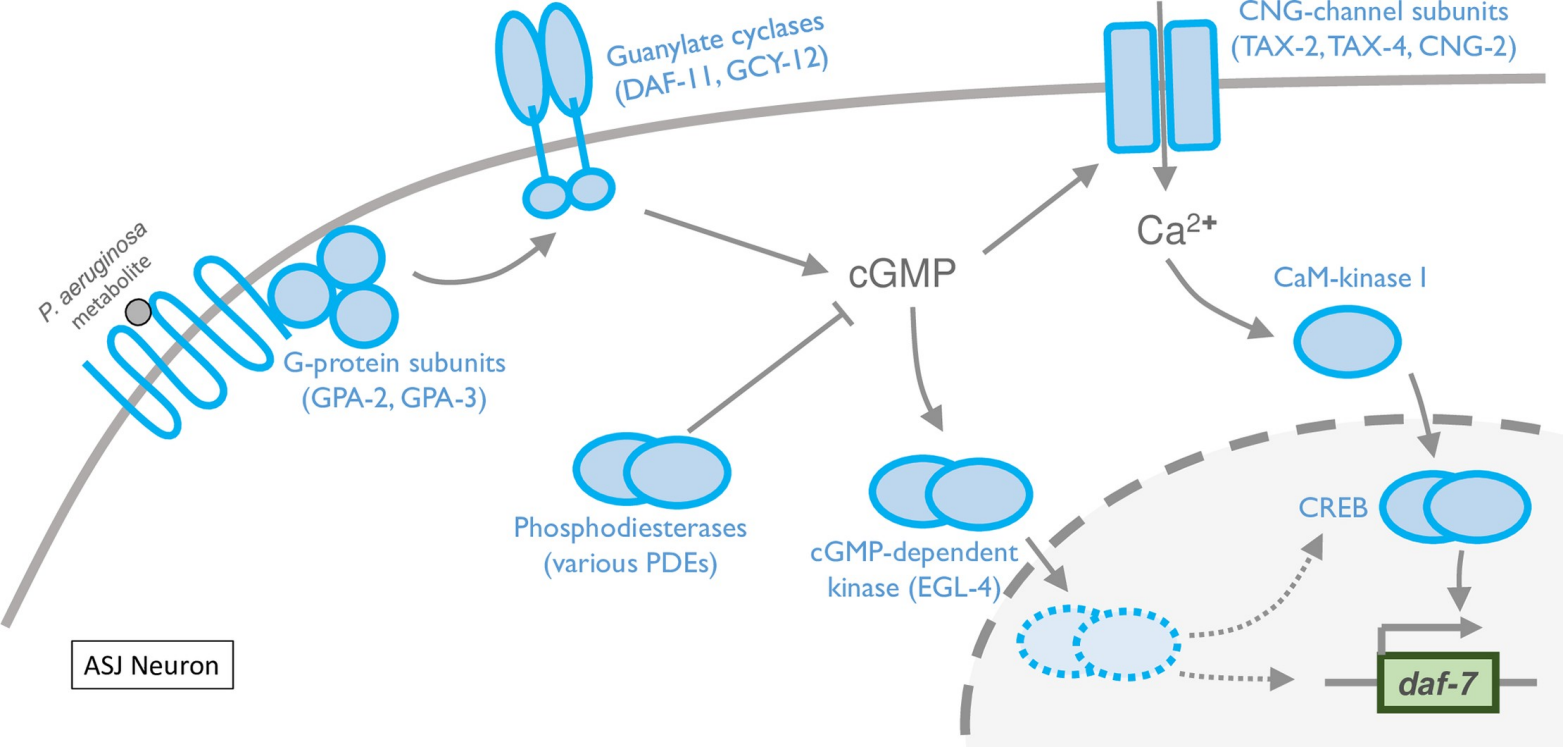
Immediate transcription of *daf-7* is selectively induced by activation of calcium-dependent and calcium-independent pathways in ASJ neurons. A schematic describing the current model for the sensory transduction pathway in the ASJ neurons resulting in fast neuronal gene transcription in response to *P*. *aeruginosa* metabolite phenazine-1-carboxamide (PCN). The model highlights the role of canonical signal transduction pathway molecules as well the added role of the cGMP-dependent kinase EGL-4 as one of the two parallel pathways required for the induction of *daf-7*. Note that the activation of both pathways are required for the full induction of *daf-7*.

The gene for DAF-7, a neuroendocrine TGF-beta ligand, is rapidly transcribed in the ASJ neurons upon exposure to *P*. *aeruginosa* metabolites [6]. The induction of *daf-7* expression in the ASJ neurons promotes increased levels of DAF-7, which act in conjunction with infection-associated modulation of internal state to promote avoidance of *P*. *aeruginosa* [7,17]. In this study, we have identified and characterized several mutants that define the signaling mechanisms coupling the sensing of *P*. *aeruginosa* metabolites to the induction of *daf-7* expression in the ASJ neuron pair. The identification of cGMP-dependent signaling proteins, CNG-2 and EGL-4, pointed to a pivotal role for cGMP. This was further corroborated by the involvement of guanylate cyclases, GCY-12 and DAF-11, the effect of inactivating multiple redundant phosphodiesterases acting on cGMP, and chemical induction of *Pdaf-7*::*gfp* expression in the ASJ neurons using pCPT-cGMP. Our data suggest a model for the sequence of cellular signaling events that are initiated by the innate recognition of *P*. *aeruginosa* metabolites and result in the rapid induction of *daf-7* expression (Fig 5). In particular, cGMP-dependent signaling through CNG-2 activates a canonical calcium-dependent signaling pathway that likely activates CREB. In parallel, cGMP-dependent signaling activates EGL-4, which functions in the nucleus in concert with calcium-dependent signaling converging on CREB. Both pathways are required for the full activation of *daf-7*, as inactivation of either pathway results in the inability to robustly upregulate *daf-7*.

TAX-2, TAX-4, and CNG-2 are subunits for the hetrotetrameric cyclic nucleotide-gated channel, and DAF-11 and GCY-12 are subunits for the dimeric guanylyl cyclase. In *tax-2*, *tax-4*, and *daf-11* mutants, *daf-7* expression is lost in both the ASI and ASJ neurons [6]. In contrast, *daf-7* expression in ASI neurons of *cng-2* and *gcy-12* mutants is relatively intact, even as *daf-7* expression in ASJ neurons is lost, suggestive of more ASJ-specific roles for CNG-2 and GCY-12 (Figs 1B–1E, 1J, 2A, S1 Fig, and S2 Fig). Neuron-specific activities of CNG-2 and GCY-12 may confer distinct biochemical properties to the CNG channels and guanylyl cyclases in different neurons. Such organization would be consistent with what is seen in other organisms: for example, the CNG channels in the rods and cones of the mammalian retina have different subunit compositions and thus have biochemical properties differentially optimized for the functions of each cell type [35].

Our data point to a key role for calcium-independent signaling through EGL-4 in the selective transcriptional activation of *daf-7* in ASJ neurons. Various external stimuli have been shown to activate ASJ neurons as measured by calcium level changes, such as low pH, salt, and *E*. *coli* supernatant [20], whereas the robust expression of *daf-7* in the ASJ neurons is activated selectively by *P*. *aeruginosa*. Loss-of-function *egl-4* mutants are unable to induce *daf-7* in ASJ neurons on *P*. *aeruginosa* (Fig 3E), and the *daf-*7 transcriptional response to the cGMP analog pCPT-cGMP is severely compromised (Fig 4C), underlining the requirement of EGL-4 in *daf-7* expression. Moreover, *egl-4* mutants have wild-type calcium influx in the ASJ neurons when exposed to phenazine-1-carboxamide (Fig 3G), implying the necessity, but not sufficiency of calcium influx in the induction of *daf-7* expression in the ASJ neurons in response to *P*. *aeruginosa*. Thus, EGL-4 activation and calcium influx seem to work together to regulate *daf-7* expression.

Calcium and cGMP have been known to work collaboratively to regulate immediate early gene expression in various neuron types [36–39]. However, our data demonstrate a key role for cGMP-dependent signaling functioning in concert with canonical calcium-dependent signaling pathways in a pair of primary sensory neurons, activated by physiological environmental ligands.

*C*. *elegans* is anatomically restricted in its neuronal system, with only 302 neurons to carry out sensation, data processing, and motor output all at once. Such constraints dictate that unlike mammalian olfactory neurons, *C*. *elegans* chemosensory neurons may have to process multiple types of stimuli in a single neuron, while retaining the ability to distinguish between them. The ASJ neurons routinely use calcium-dependent signaling to mediate signal transduction to a wide variety of stimuli, but our data suggest that select stimuli such as secondary pathogen metabolites can be distinguished and linked to gene transcription by engaging calcium-independent PKGs in addition to calcium-dependent signals (Fig 5). Whereas how PKGs can be activated selectively in response to different stimuli remains to be explored further, our data provide an indication of how transcriptional responses in sensory neurons of *C*. *elegans* may be gated through distinct signal transduction pathways to result in selective changes in gene expression to promote adaptive behaviors.

## Materials and methods

### *C*. *elegans* Strains

All animals were maintained and fed as previously described [40]. The animals were incubated at 20°C unless any of the strains were considered temperature-sensitive, in which case they were grown at 16°C. Please see S1 Table for a complete list of strains used in this study.

### *Pdaf-7*::*gfp* induction assays

For experiments quantifying the level of *Pdaf-7*::*gfp* on the *Pseudomonas aeruginosa* strain PA14, bacteria was cultured overnight in 3 mL LB broth at 37°C, and the following day 7 μl was seeded onto 3.5cm slow-killing assay (SKA) plates as described previously [15]. The seeded plates were maintained at 37°C overnight and then transferred to room temperature, where they were kept additional two days before experiments. To preemptively rid animals of bacterial contamination, gravids were bleached to get a large amount of eggs. Animals were loaded onto PA14 at stage L4 and then were kept at 25°C for 14–16 hours before quantification. For assays using pCPT-cGMP, pCPT-cGMP was added to SKA plates in mixed DMSO and water, with the resulting concentration in plates to be 5 mM. Plates were left overnight for the chemical to diffuse. The next day, 5 μl inoculate of *E*. *coli* strain OP50 was seeded to the middle, and plates were kept in room temperature overnight before experiments commenced. Animals were similarly egg-prepped for this condition as noted above. L4s were loaded onto the center of the SKA plates and kept at 20°C for 17–20 hrs before quantification.

### Imaging and quantification of *Pdaf-7p*::*gfp* levels

Animals were mounted on glass slides with agarose pads and anesthetized with 50 mM sodium azide. Animals were imaged using a Zeiss Axioimager Z1. Quantification of GFP brightness was conducted with FIJI by obtaining maximum fluorescence values within the ASJ, or ASI neurons. Y-axes are denoted by arbitrary brightness units (A.B.U.). All imaging for pictures were conducted on the Zeiss Axioimager Z1, except imaging of animals containing the mCherry-EGL-4(ΔNLS) construct, which was conducted on the Zeiss LSM800 confocal microscope.

### Generation of transgenic animals

The *trx-1* promoter (1.1 kb) was amplified by PCR from genomic DNA [41], and *unc-54* 3’ UTR was amplified from Fire Vector pPD95.75. *cng-2* cDNA generously provided by P Sengupta, *gcy-12* cDNA by M. Fujiwara, *egl-4* cDNA by N. D. L’Etoile, and *crh-1* cDNA by C. T. Murphy, were all respectively amplified by PCR. Finally, the *trx-1* promoter, respective cDNAs, and the *unc-54* 3’ UTR were cloned into plasmids using NEBuilder HiFi DNA Assembly (New England Biolabs, Ipswich, MA). The plasmids were microinjected at 40–50 ng/μl concentration, along with *ofm-1p*::*gfp* as a co-injection marker at 30–40 ng/μl for ASJ specific expression. For generation of strains with calcium indicators, amplified *trx-1* promoter was fused with GCaMP5G to express the indicator in ASJ neurons only.

### Calcium imaging

The animals were immobilized and exposed to soluble compound in a controlled manner using a microfluidics chip as previously described [42]. Imaging was carried out at 40x with a Zeiss Axiovert S100 inverted microscope equipped with an Andor iXon EMCCD camera. Stimuli were given at noted concentrations. Chemicals were dissolved in DMSO for stock storage, and they were further diluted in S-Basal medium for experiments. Phenazine-1-carboxamide was obtained from Princeton BioMolecular Research (Princeton, NJ). Both mutant and control animals were imaged on the same day and animals were tested in an alternating manner (e.g. wild-type, mutant, wild-type, mutant, etc.) to minimize bias. Data analysis was done with a custom MATLAB script written by Nikhil Bhatla.

## Supporting information

S1 Fig *daf-7* is induced in ASI neurons of *cng-2* mutants upon exposure to *P. aeruginosa* to a similar degree seen in wild-type. Pdaf*-7*::*gfp* levels of ASI neurons in *cng-2* mutants after exposure to *P*. *aeruginosa*. All error bars indicate standard deviation. n.s. indicates p > 0.05 by Mann-Whitney U test.
(TIF)

S2 Fig *daf-7* is induced in ASI neurons of *gcy-12* mutants upon exposure to *P. aeruginosa* to levels similar to or exceeding that of wild-type. *Pdaf-7*::*gfp* levels of ASI neurons in *gcy-12* mutants after exposure to *P*. *aeruginosa*. All error bars indicate standard deviation. **p < 0.01, *p < 0.05 by Mann-Whitney U test.
(TIF)

S3 Fig ASJ *daf-7* transcription in phosphodiesterase mutants is further elevated upon exposure to *P. aeruginosa*. *Pdaf-7*::*gfp* expression before and after exposure to *P*. *aeruginosa* for the quadruple phosphodiesterase mutant. All error bars indicate standard deviation. ****p < 0.0001 by Mann-Whitney U test.
(TIF)

S4 Fig NaCl does not induce *daf-7* transcription in the ASJ neurons. *Pdaf-7*::*gfp* expression after being transferred to plates containing the indicated NaCl concentration, or *P*. *aeruginosa* (PA14) as control. Animals were transferred as L4s and were imaged 17 hours later. All error bars indicate standard deviation. ****p < 0.0001 by Mann-Whitney U test.
(TIF)

S5 Fig The pASJ::mCherry::ΔNLS-EGL-4 construct is not visibly excluded from the nucleus. (A-D) Each column represents the same image, split into different channels. Top channel visualizes Pdaf-7::gfp, which outlines the ASJ cells. Bottom channel visualizes pASJ::mCherry::ΔNLS-EGL-4, which contains a modified egl-4 cDNA with an ablation of the predicted NLS (JI Lee et al., 2010). Although a minority of animals exhibited somewhat nuclear-excluded mCherry (panel B), majority of animals showed a diffuse pattern of mCherry throughout the ASJ cells. We also observed that some individual animals showed substantial aggregation of the mCherry construct, making it difficult to interpret localization patterns (not shown).
(TIF)

S6 Fig Individual ΔF/F0 calcium level traces. (A, B) Individual traces underlying main Fig 1N and 1O. (C, D) Individual traces underlying main Fig 3F and 3G.
(TIF)

S1 Table All strains used in this study. (PDF)
