# Supplementary figures and images for: Immediate activation of chemosensory neuron gene expression by bacterial metabolites is selectively induced by distinct cyclic GMP-dependent pathways in Caenorhabditis elegans
Source: PLoS Genet. 2020 Aug 10;16(8):e1008505. doi: 10.1371/journal.pgen.1008505 (PMC7416920; doi:10.1371/journal.pgen.1008505)

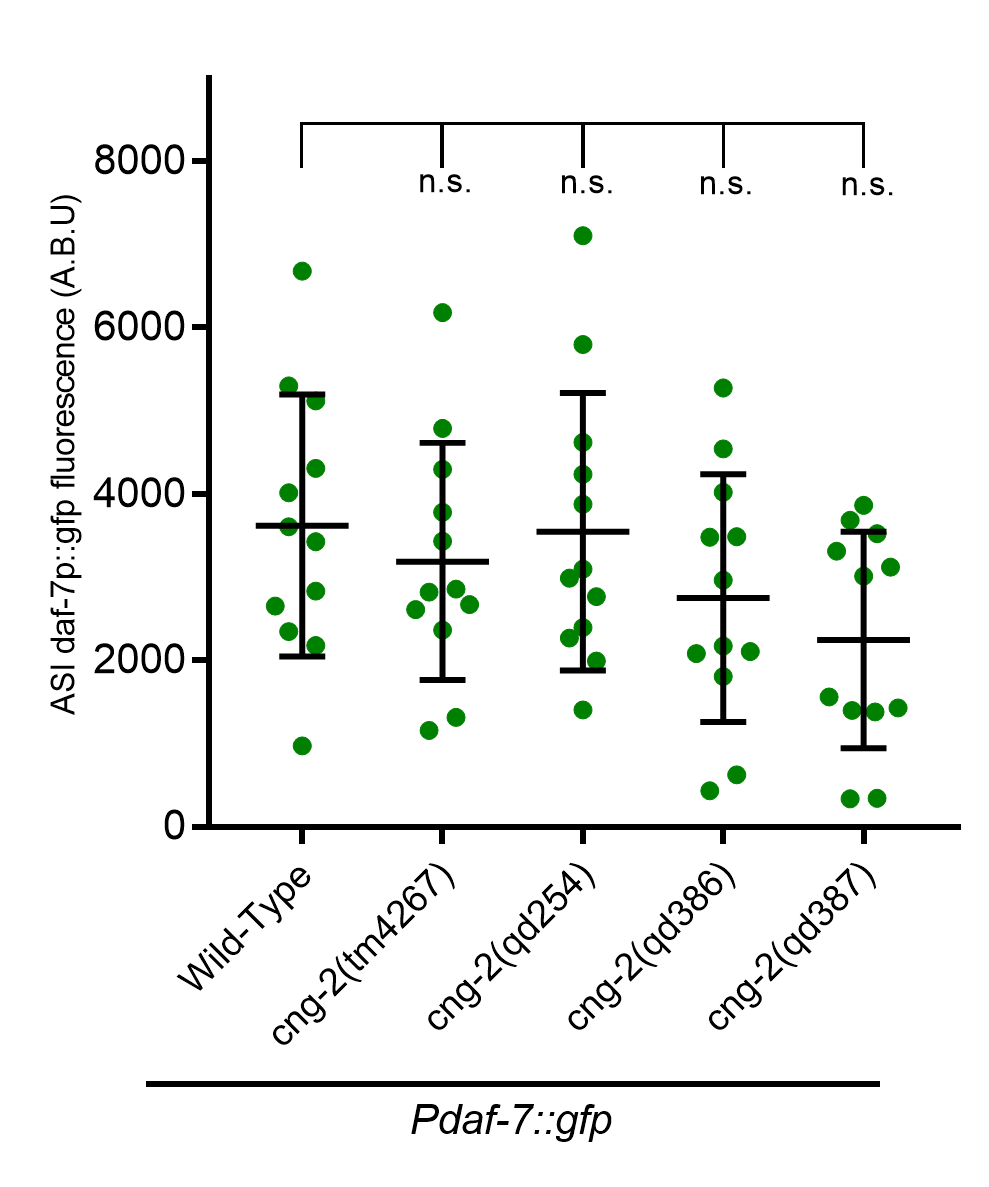

Supplement: S1 Fig — Pdaf-7::gfp levels of ASI neurons in cng-2 mutants after exposure to P. aeruginosa. All error bars indicate standard deviation. n.s. indicates p > 0.05 by Mann-Whitney U test. (TIF) [file pgen.1008505.s001.tif]

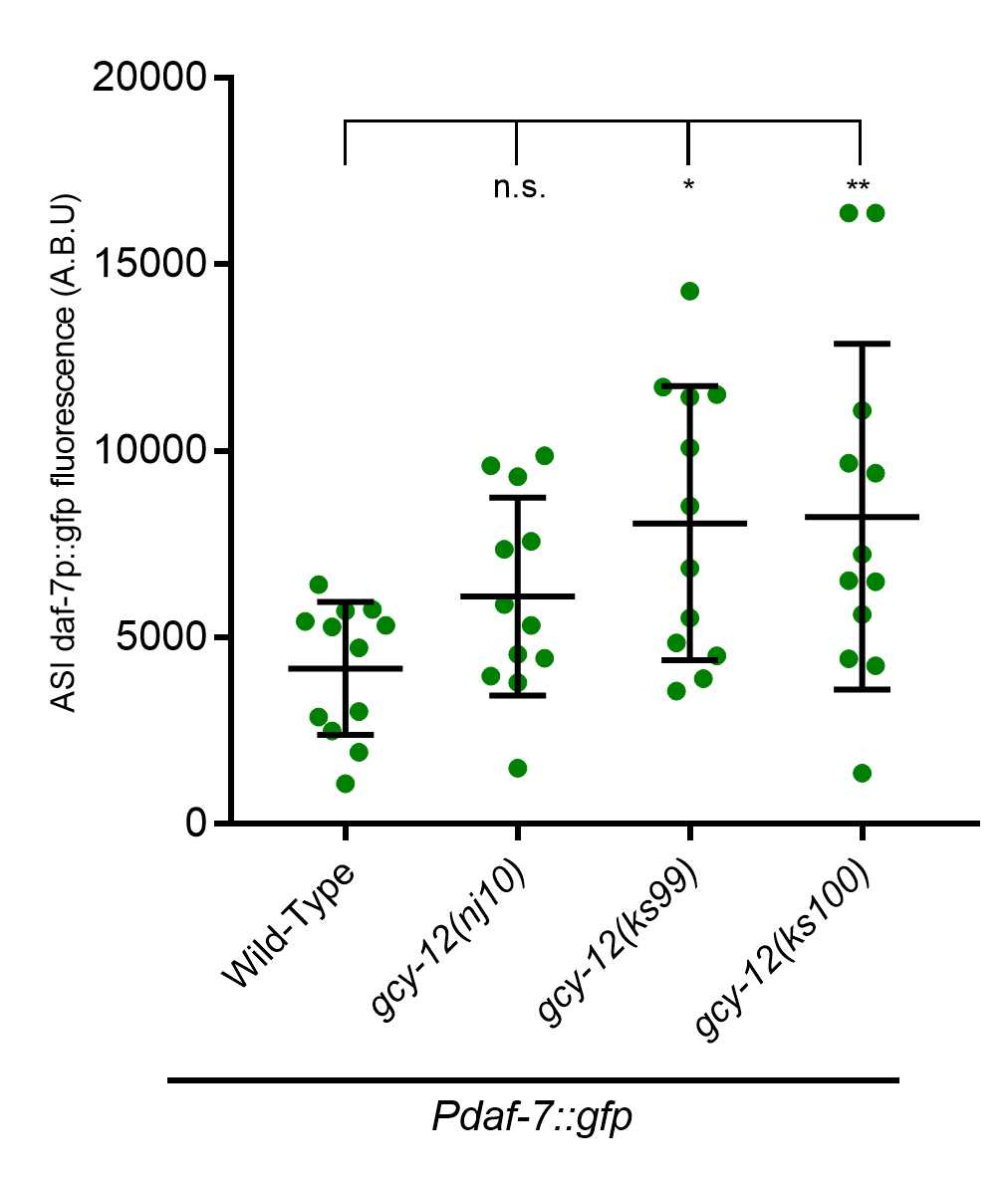

Supplement: S2 Fig — Pdaf-7::gfp levels of ASI neurons in gcy-12 mutants after exposure to P. aeruginosa. All error bars indicate standard deviation. **p < 0.01, *p < 0.05 by Mann-Whitney U test. (TIF) [file pgen.1008505.s002.tif]

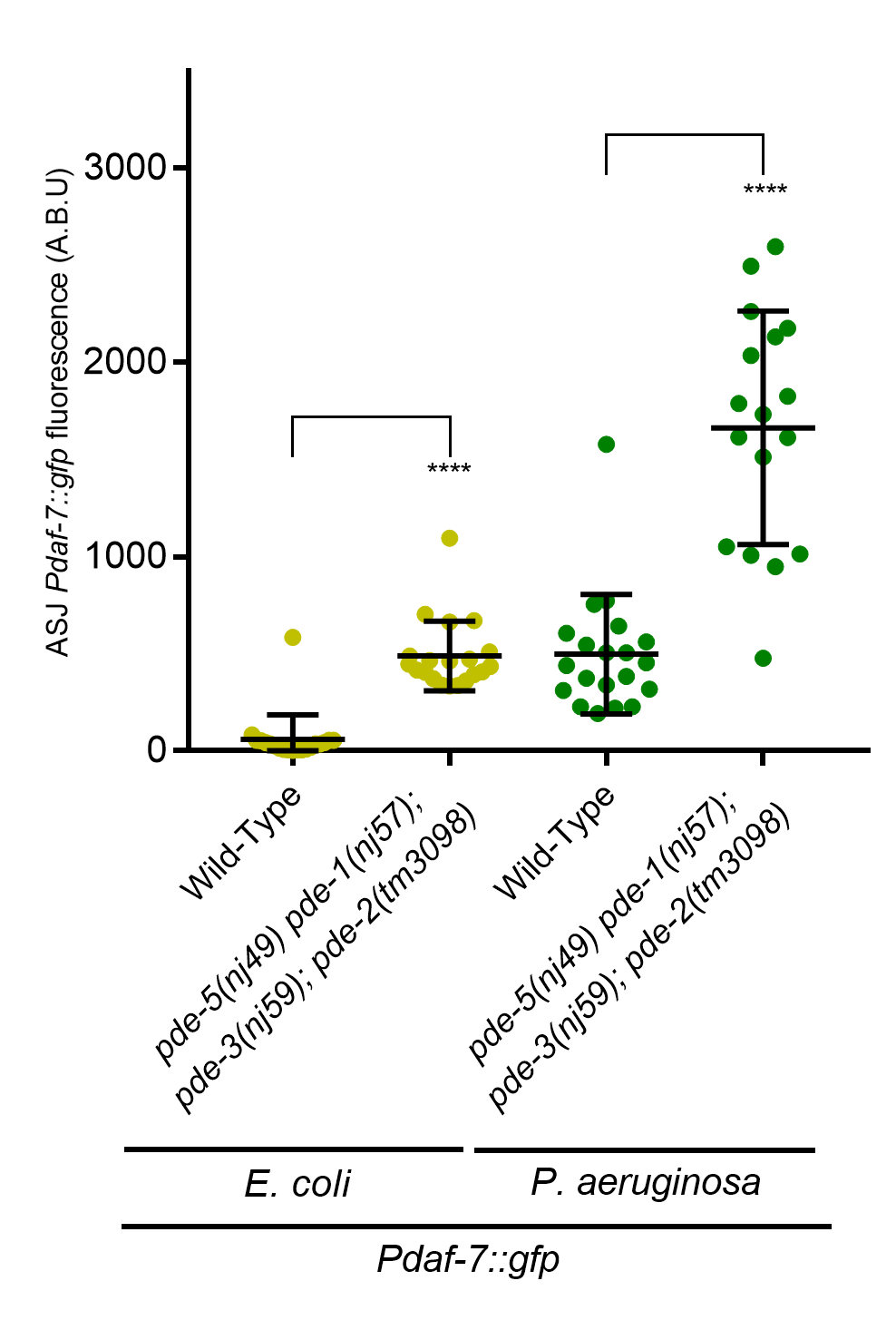

Supplement: S3 Fig — Pdaf-7::gfp expression before and after exposure to P. aeruginosa for the quadruple phosphodiesterase mutant. All error bars indicate standard deviation. ****p < 0.0001 by Mann-Whitney U test. (TIF) [file pgen.1008505.s003.tif]

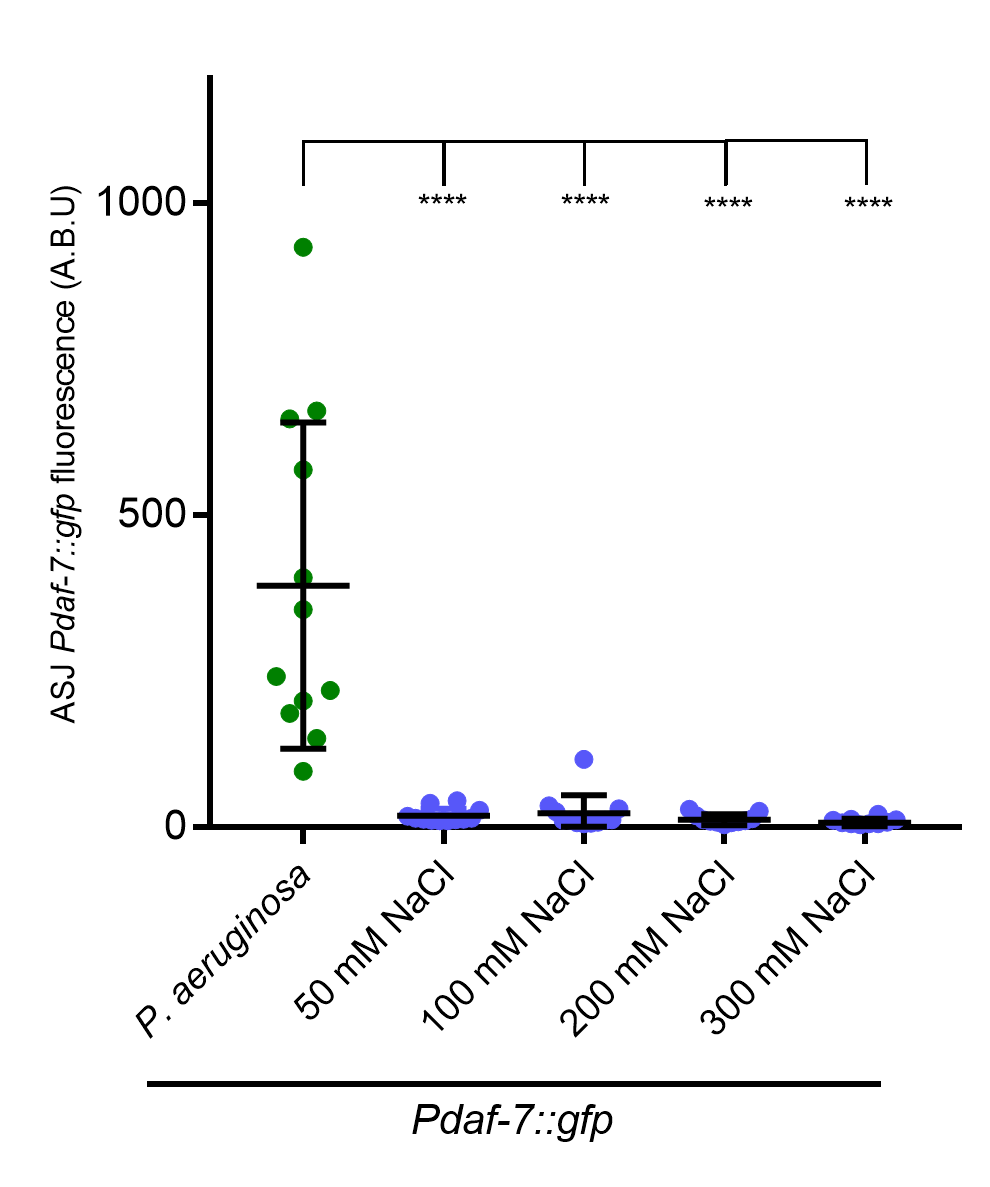

Supplement: S4 Fig — Pdaf-7::gfp expression after being transferred to plates containing the indicated NaCl concentration, or P. aeruginosa (PA14) as control. Animals were transferred as L4s and were imaged 17 hours later. All error bars indicate standard deviation. ****p < 0.0001 by Mann-Whitney U test. (TIF) [file pgen.1008505.s004.tif]

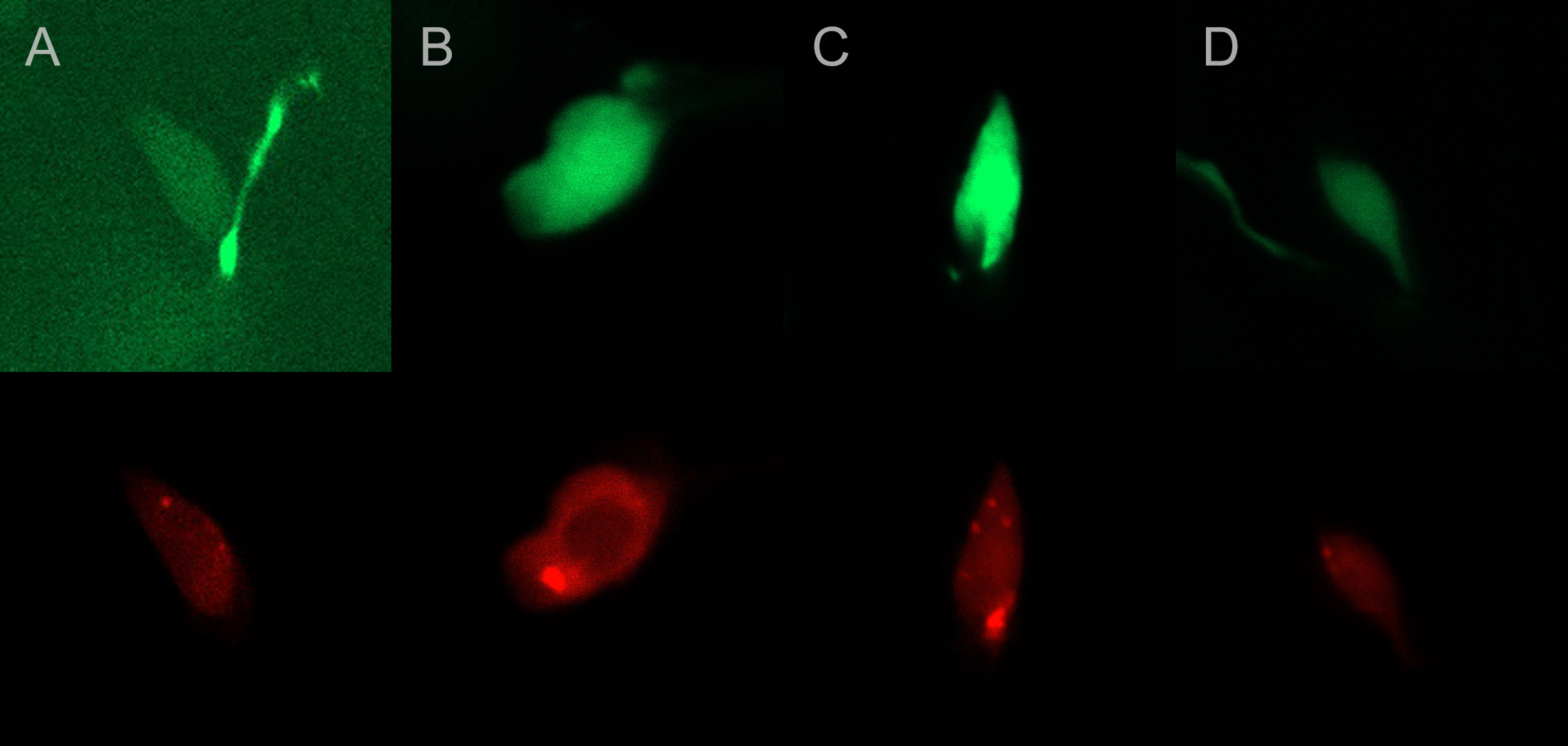

Supplement: S5 Fig — (A-D) Each column represents the same image, split into different channels. Top channel visualizes Pdaf-7::gfp, which outlines the ASJ cells. Bottom channel visualizes pASJ::mCherry::ΔNLS-EGL-4, which contains a modified egl-4 cDNA with an ablation of the predicted NLS (JI Lee et al., 2010). Although a minority of animals exhibited somewhat nuclear-excluded mCherry (panel B), majority of animals showed a diffuse pattern of mCherry throughout the ASJ cells. We also observed that some individual animals showed substantial aggregation of the mCherry construct, making it difficult to interpret localization patterns (not shown). (TIF) [file pgen.1008505.s005.tif]

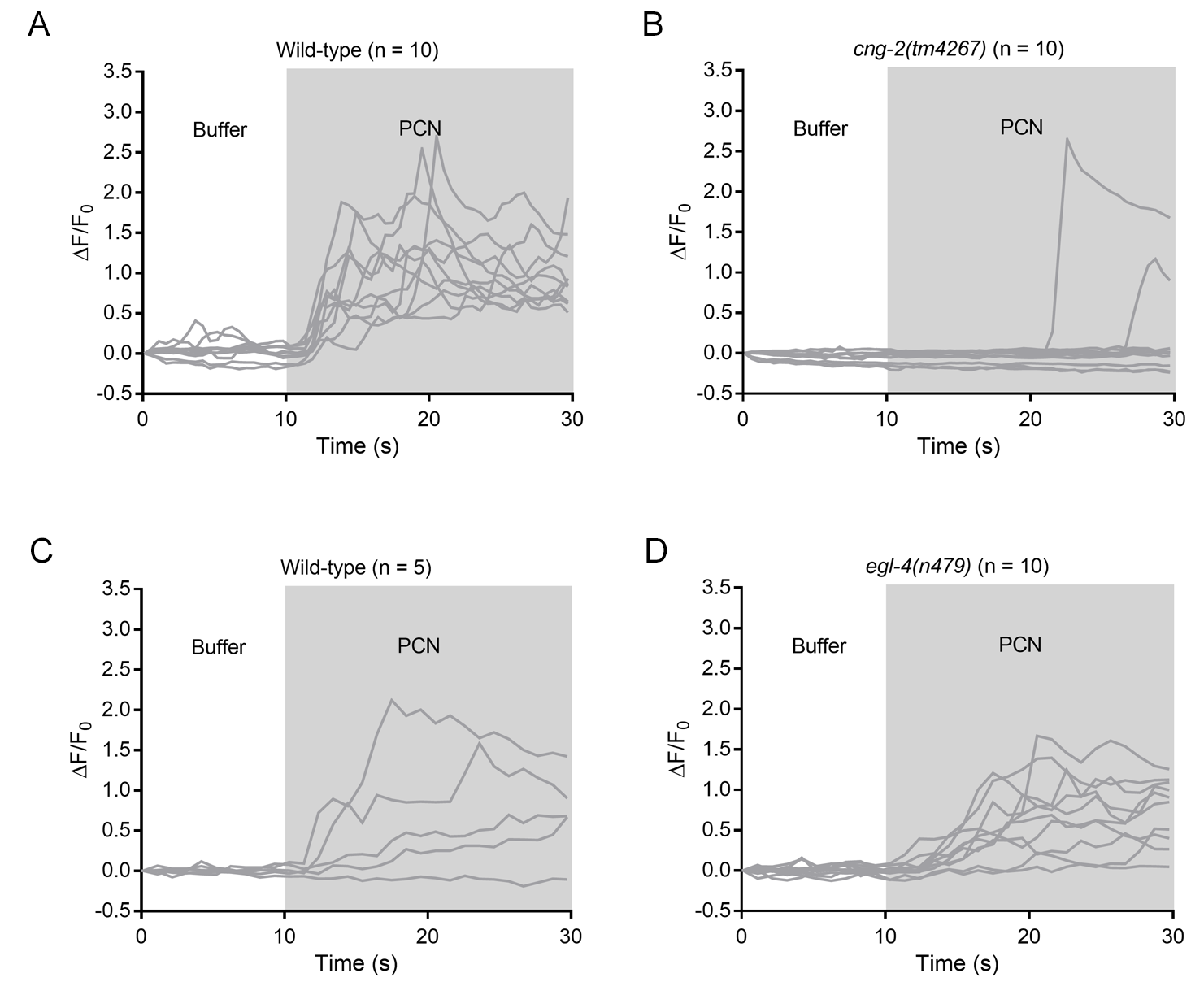

Supplement: S6 Fig — (A, B) Individual traces underlying main Fig 1N and 1O. (C, D) Individual traces underlying main Fig 3F and 3G. (TIF) [file pgen.1008505.s006.tif]
