# Supplementary material for: Immediate activation of chemosensory neuron gene expression by bacterial metabolites is selectively induced by distinct cyclic GMP-dependent pathways in Caenorhabditis elegans
Source: PLoS Genet. 2020 Aug 10;16(8):e1008505. doi: 10.1371/journal.pgen.1008505 (PMC7416920; doi:10.1371/journal.pgen.1008505)
Supplement: S1 Table — (PDF) [file pgen.1008505.s007.pdf]

S1 Table. All strains used in this study

STRAIN	GENOTYPE
N2	Wild-Type
FK181	*ksIs2[Pdaf-7::GFP + rol-6(su1006)]*
ZD727	*ksIs2; crh-1(tz2)*
ZD899*	*ksIs2; cng-2(tm4267)*
ZD882	*ksIs2; cng-2(qd254)*
ZD887	*ksIs2; gpa-2(pk16) gpa-3(pk35)*
ZD1048	*ksIs2; cmk-1(oy21)*
ZD1401	*ksIs2; gcy-12(nj10)*
ZD1922	*ksIs2; egl-4(n478); him-5(e1490)*
ZD1737	*qdEx136[trx-1p::GCaMP5; ofm-1::gfp]*
ZD1949	*ksIs2; egl-4(ad450); him-5(e1490)*
ZD2020	*cng-2(tm4267); qdEx136[trx-1p::GCaMP5; ofm-1p::gfp]*
ZD2090	*ksIs2; gcy-12(ks100)*
ZD2091	*pde-5(nj49) ksIs2*
ZD2105	*pde-5(nj49) pde-1(nj57) ksIs2*
ZD2150	*pde-1(nj57) ksIs2*
ZD2151	*pde-5(nj49) pde-1(nj57) ksIs2; pde-3(nj59); pde-2(tm3098)*
ZD2170	*egl-4(n479); qdEx136[trx-1p::GCaMP5::unc-54 5'UTR; ofm-1p::gfp]*
ZD2534	*ksIs2; cng-2(qd386)*
ZD2535	*ksIs2; cng-2(qd387)*
ZD2250	*ksIs2; cng-2(qd254); qdEx[pJP3(trx-1p::cng-2cDNA::unc-54UTR) + ofm-1p::gfp] Line #1*
ZD2251	*ksIs2; cng-2(qd254); qdEx[pJP3(trx-1p::cng-2cDNA::unc-54UTR) + ofm-1p::gfp] Line #2*
ZD2275	*ksIs2; egl-4(n479ts)*
ZD2283	*ksIs2; egl-4(ad450) cng-2(qd254)*
ZD2307	*ksIs2; egl-4(n479); qdEx[trx-1p::mCherry::egl-4cDNA + ofm-1p::gfp] Line #1*
ZD2308	*ksIs2; egl-4(n479); qdEx[trx-1p::mCherry::egl-4cDNA + ofm-1p::gfp] Line #2*
ZD2526	*ksIs2; gcy-12(ks100); qdEx[trx-1p::gcy-12cDNA::unc-54UTR + ofm-1p::gfp] Line #1*
ZD2527	*ksIs2; gcy-12(ks100); qdEx[trx-1p::gcy-12cDNA::unc-54UTR + ofm-1p::gfp] Line #2*
ZD2536	*ksIs2; egl-4(n479); qdEx[pJP17(trx-1p::SV40-nls::mCherry::egl-4cDNA::unc54UTR) + ofm-1p::gfp] Line #1*
ZD2537	*ksIs2; egl-4(n479); qdEx[pJP17(trx-1p::SV40-nls::mCherry::egl-4cDNA::unc54UTR) + ofm-1p::gfp] Line #2*
ZD2538	*ksIs2; egl-4(n479); qdEx[pJP17(trx-1p::SV40-nls::mCherry::egl-4cDNA::unc54UTR) + ofm-1p::gfp] Line #3*
ZD2545	*ksIs2; crh-1(tz2); qdEx[pJP13(trx-1p::crh-1cDNA::unc-54UTR) + ofm-1p::gfp] Line #1*

ZD2546	*ksIs2; crh-1(tz2); qdEx[pJP13(trx-1p::crh-1cDNA::unc-54UTR) + ofm-1p::gfp] Line #2*
ZD2547	*ksIs2; crh-1(tz2); qdEx[pJP13(trx-1p::crh-1cDNA::unc-54UTR) + ofm-1p::gfp] Line #3*
ZD2598	*ksIs2; egl-4(n479); qdEx[trx-1p::mCherry::egl-4cDNA(T276A,T400A)::unc-54UTR) + ofm-1p::gfp] Line #1*
ZD2599	*ksIs2; egl-4(n479); qdEx[trx-1p::mCherry::egl-4cDNA(T276A,T400A)::unc-54UTR) + ofm-1p::gfp] Line #2*
ZD2600	*ksIs2; egl-4(n479); qdEx[trx-1p::mCherry::egl-4cDNA(T276A,T400A)::unc-54UTR) + ofm-1p::gfp] Line #3*

**tm4267* is currently classified as lethal or sterile by the National BioResource Project of Japan and was originally maintained on a balancer. However, *cng-2(tm4267)* is viable, as we were able to backcross away the linked lethal mutation.
